# Delayed Seasonality but Consistent Age Burden: RSV Epidemiology Pre–, During, and Post–COVID-19 Pandemic on the Kenyan Coast (2008–2026)

**DOI:** 10.1093/ofid/ofag600

**Published:** 2026-09-22

**Authors:** Esther Nyadzua Katama, Grace Maina, Martin Mutunga, Clement Lewa, Robinson Cheruiyot, Agustus Kea, Mohammed Shebe, Joyce U Nyiro, Charles J Sande, Charles N Agoti

**Affiliations:** Kenya Medical Research Institute (KEMRI) - Wellcome Trust Research Programme (KWTRP), P.O. Box 230-80108, Kilifi, Kenya; Kenya Medical Research Institute (KEMRI) - Wellcome Trust Research Programme (KWTRP), P.O. Box 230-80108, Kilifi, Kenya; Kenya Medical Research Institute (KEMRI) - Wellcome Trust Research Programme (KWTRP), P.O. Box 230-80108, Kilifi, Kenya; Kenya Medical Research Institute (KEMRI) - Wellcome Trust Research Programme (KWTRP), P.O. Box 230-80108, Kilifi, Kenya; Kenya Medical Research Institute (KEMRI) - Wellcome Trust Research Programme (KWTRP), P.O. Box 230-80108, Kilifi, Kenya; Kenya Medical Research Institute (KEMRI) - Wellcome Trust Research Programme (KWTRP), P.O. Box 230-80108, Kilifi, Kenya; Kenya Medical Research Institute (KEMRI) - Wellcome Trust Research Programme (KWTRP), P.O. Box 230-80108, Kilifi, Kenya; Kenya Medical Research Institute (KEMRI) - Wellcome Trust Research Programme (KWTRP), P.O. Box 230-80108, Kilifi, Kenya; Kenya Medical Research Institute (KEMRI) - Wellcome Trust Research Programme (KWTRP), P.O. Box 230-80108, Kilifi, Kenya; Centre for Tropical Medicine and Global Health, University of Oxford, Oxford, UK; Kenya Medical Research Institute (KEMRI) - Wellcome Trust Research Programme (KWTRP), P.O. Box 230-80108, Kilifi, Kenya; Department of Public Health, Pwani University, Kilifi, Kenya

**Keywords:** COVID-19, Hospitalization, Kenya, RSV, seasonality

## Abstract

**Background:**

The COVID-19 pandemic and associated nonpharmaceutical interventions disrupted the established epidemiological patterns of many endemic respiratory viruses globally. However, its impact on respiratory syncytial virus (RSV) epidemiology in sub-Saharan Africa is understudied.

**Methods:**

We analyzed data from a continuous hospital-based pediatric pneumonia surveillance program in coastal Kenya January 2008–June 2026. Children ≤5 years, admitted with World Health Organization–defined syndromic severe or very severe pneumonia were tested for RSV using quantitative reverse transcriptase polymerase chain reaction. The period was stratified into prepandemic (January 2008–March 2020), during pandemic (March 2020–May 2023), and postpandemic (May 2023–June 2026). Multivariable logistic regression evaluated clinical and demographic shifts across periods.

**Results:**

Among 10 374 tested children, 2442 (23.5%) were RSV positive. Positivity declined from 25.3% (1919/7585) prepandemic to 18.3% (235/1282) during the pandemic and 19.1% (288/1507) postpandemic. Overall median age of RSV-positive patients remained statistically consistent across all periods (4.3 vs 3.7 vs 3.1 months; *P*  *= .108*), with ≤6-month-olds accounting for ∼62% of admissions throughout. However, in the 2020–2021 season, RSV-positive children had increased median age (6.7; interquartile range: 2.0–15.1 months). In multivariable analysis, fever was less frequent during and postpandemic (adjusted *P*  *<* .001), and cough decreased postpandemic (adjusted *P* = .002). Pneumonia severity and overall mortality did not significantly differ across the periods. Local RSV season onsets exhibited 1–2 months’ delay during and postpandemic compared to prepandemic.

**Conclusions:**

Despite a repeated 1- to 2-month delay of RSV season onsets on the Kenyan coast and minor clinical symptoms shifts, the core demographic patterns remained stable. The persisting severe RSV disease in <6-month-olds highlights this population for RSV interventions.

## Teaser

A longitudinal RSV surveillance study on the Kenyan coast reveals post–COVID-19 pandemic, there has been a sustained 1- to 2-month delay in RSV epidemic onset and minor clinical symptoms shifts, but the core burden of severe RSV disease remains in the ≤6-month-olds.

## BACKGROUND

Human respiratory syncytial virus (RSV) is a leading cause of acute respiratory tract infections worldwide and a primary driver of pediatric hospitalizations [1]. RSV infection clinical spectrum ranges from asymptomatic infection status to mild upper respiratory tract illness to severe or fatal pneumonia and bronchiolitis [2–4]. Because of its substantial clinical presentation overlaps with other cocirculating respiratory pathogens, a laboratory test is required to confirm RSV infection [5–7]. Globally, RSV is estimated to be responsible for approximately 101 400 deaths (95% confidence interval [CI]: 84 500–125 200) in children aged <5 years annually [8], with >90% of these fatalities occurring in low- and middle-income countries [8]. Notably, approximately half of these deaths occur in highly vulnerable infants aged ≤6 months old [9–11].

Historically, RSV exhibits consistent seasonal transmission cycles in communities, typically peaking during winter in temperate climatic regions [12], but has a less clear link to weather patterns in tropical and subtropical areas like Kenya [13, 14]. The emergence of severe acute respiratory syndrome coronavirus 2 (SARS-CoV-2) in late 2019, the etiological agent of coronavirus disease 2019 (COVID-19) [15], and the widespread implementation of associated non-pharmaceutical interventions (NPIs) disrupted established seasonal patterns of many endemic respiratory viruses globally [16–20]. The relaxation of these measures was followed by unusual RSV resurgences, characterized by altered epidemic timing and shifting of severe disease age demographic patterns [21–23]. Several high-income countries reported RSV severe disease surges in older pediatric populations, hypothesized to be a result of an “immunity debt” resulting from extended periods of reduced virus transmission [24, 25]. However, it remains unclear whether the clinical presentation of RSV changed during or after the COVID-19 pandemic across different global settings [18, 26].

Although the post–COVID-19 pandemic dynamics have been documented in many high-income settings [16, 27, 28], the specific impact on RSV seasonality, age distribution, and disease severity remains understudied in sub-Saharan Africa [29, 30]. Understanding whether pandemic-induced shifts in RSV epidemiology are transient or permanent is essential for optimizing local RSV disease control measures, for example, the newly licensed, transiently acting interventions such as long-acting monoclonal antibodies (Nirsevimab and Clesrovimab) and maternal vaccine (Abrysvo) [31]. These interventions may require precise seasonal or prenatal administration for maximum efficacy [32, 33]. To address this gap, we leveraged our longitudinal 19-seasons RSV surveillance platform at the Kenyan Coast to compare the age distribution, symptoms profile, disease severity and seasonal patterns of RSV cases detected pre-, during, and post–COVID-19 pandemic periods in the region (2008–2026).

## MATERIALS AND METHODS

### Study Design, Setting, and Population

We leveraged the continuous hospital-based paediatric pneumonia surveillance at Kilifi County Referral Hospital (KCRH) on the Kenyan coast between 1 January 2008 and 30 June 2026 [9, 34, 35]. Coordinated by the Kenya Medical Research Institute (KEMRI)-Wellcome Trust Research Programme (KWTRP), the surveillance aimed to monitor local epidemiological patterns and pathogen-specific disease burdens, providing suitable baseline data for subsequent vaccine studies [9, 35]. Eligible participants included all children aged younger than 60 months who were admitted with World Health Organization–defined syndromic severe or very severe pneumonia. Severe pneumonia was defined as history of cough or difficulty breathing accompanied by chest indrawing. Very severe pneumonia was defined as history of cough or difficulty breathing and at least one of the following danger signs: inability to feed, prostration, unconsciousness, or oxygen saturation of <90% by fingertip pulse oximetry [34, 36]. This included bronchiolitis cases. Notably, health worker strikes interrupted the surveillance 14 times between 5 and 13 December 2011 and 12 to 23 March 2026 [37], (Supplementary Table 1).

### Clinical Procedures and Data Collection

Upon admission, demographic details (e.g., sex, date of birth, location) and clinical data (e.g., vital measurements, signs and symptoms, medical history, nutritional status) were collected from the participant or their caregiver using a standard electronic questionnaire. Attending clinicians recorded presenting signs and symptoms, including cough, fever (above 37.8 °C), vomiting, diarrhea, crackles, wheezing and chest indrawing, among others. Consenting participants subsequently provided a respiratory specimen, a nasopharyngeal and oropharyngeal swab, for pathogen detection within 48 hours of admission.

### Laboratory Methods

RSV detection was undertaken by both antigen detection and nucleic acid detection. For the latter, briefly, viral nucleic acids were extracted from the respiratory samples using QIAamp Viral RNA Mini-kit (Qiagen, UK). These were then tested for 15 different respiratory pathogen targets including RSV-A and RSV-B using multiplex quantitative reverse transcriptase polymerase chain reaction (RT-PCR [qPCR]) [38]. The RSV primers and probes included in the assay distinguish RSV-A and RSV-B and have been described elsewhere [38]. Whenever the positivity by the antigen and molecular detection diverged, discrepant results were further investigated (Supplementary Figure 4, Supplementary Table 5). The RSV-B assay was updated twice during the study period because of an observed decline in its performance caused in part by emergence of mismatches in the reverse primer relative to the sequences of the circulating strains. The first update was implemented in July 2016 [39] and the second in January 2025 (Supplementary Table 2). A sample was considered RSV-A or RSV-B positive if it returned a qPCR cycle threshold (Ct) value of ≤35.0 and the run controls passed.

### Epidemiological Definitions

To evaluate the impact of the pandemic, the study period was stratified into 3 distinct phases: (1) “prepandemic” (1 January 2008 to 12 March 2020), (2) “during pandemic” (13 March 2020 to 5 May 2023), and (3) “postpandemic” (6 May 2023 to 30 June 2026). Note that Kenya detected the first case of COVID-19 on 13 March 2020 [40] and the World Health Organization declared the end of the COVID-19 Public Health Emergency of International Concern on 5 May 2023 [41]. An epidemic was considered to have commenced when either: (1) at least 10% of tested samples were positive for RSV or (2) at least 2 laboratory-confirmed RSV cases were reported per week for 2 consecutive weeks. RSV epidemic periods were defined empirically as previously described [35]. Epidemic periods were considered ongoing if at least 1 of these criteria was met, with the requirement that an epidemic lasted for a minimum of 4 consecutive weeks [35, 42–44]. Epidemic offset was defined as the final consecutive week meeting these criteria before falling below threshold levels. The predominant RSV subgroup was defined as the subgroup representing ≥55% of laboratory-confirmed RSV infections within each RSV epidemic.

### Statistical Analysis

Demographic characteristics and clinical presentations were compared between RSV-positive and RSV-negative cases, as well as across the three study phases (prepandemic, during pandemic, and postpandemic). Summary statistics on distribution of RSV cases by month, age, and clinical symptoms were computed and assessed using the Mann–Whitney *U* test, Fisher exact test, or Pearson chi-squared test as appropriate. Epidemic onset timing, peak timing, and duration medians were compared across the study periods using Kruskal–Wallis tests. To assess whether clinical presentation of RSV-positive children changed across prepandemic, during pandemic, and postpandemic periods, multivariable logistic regression models were fitted separately for each clinical symptom. Each symptom was modelled as a function of the 3 study periods (prepandemic, during pandemic, and postpandemic) adjusting for sex, age, and RSV subgroup (RSV-A, RSV-B, and RSV-A + B) as in the formula:


$$\log\;\left(\frac{P(Y_{i}=1)}{1-P(Y_{i}=1)}\right)=\beta_{0}+\beta_{1}X_{1i}+\beta_{2}X_{2i}+\beta_{3}X_{3i}+\beta_{4}X_{4i}$$

where:

-
$Y_{i}$ = Status of a given clinical symptom for child *i* (1 = presence, 0 = absence)
-
$X_{1i}$ = Period (pre-, during, and postpandemic where prepandemic is the reference category).
-
$X_{2i}$ = Age in months.
-
$X_{3i}$ = Sex (male = 1, female = 0).
-
$X_{4i}$ = RSV subgroup (RSV-A, RSV-B, and RSV A + B coinfection where RSV-A is the reference category).

Adjusted odds ratios (aORs) with 95% CIs were estimated with *P*  *<* .05 considered as statistically significant. To account for multiple testing across symptom-specific analyses, *P* values for the study phases (pre-, during, and postpandemic) variables were adjusted using the Benjamini–Hochberg false discovery rate formula:


$$p_{\left(i\right)}^{BH}=\underset{\;j\geq i}{\min}\;\left\{\frac{m}{j}p_{\left(j\right)}\right\}$$

where:

-
$p_{\left(i\right)}^{BH}$ = The Benjamini–Hochberg–adjusted *P* value for the *i*th ordered test
-
$p_{\left(j\right)}$ = the *j*th smallest observed *P* value
- *j* = the rank of the observed *P* value
- *m* = the total number of hypothesis tests

Adjusted *P* values were used to assess statistical significance.

To assess the impact of the health care worker strikes on the surveillance, enrollment numbers, and positivity rates during strike intervals were compared with historical calendar windows using a 2-sample test of proportions (Supplementary Table 1). All data processing and statistical analyses were conducted using R version 4.6.0.

## RESULTS

### Study Population and Baseline Characteristics

The study participant flow has been summarized in Supplementary Figure 1. Between 1 January 2008 and 30 June 2026, a total of 15 211 eligible participants were admitted to KCRH. Of these, 10 374 (68.2%) participants were tested for RSV. Reasons for nontesting among the 4837 eligible participants included lack of consent (n = 2255), unknown reason (n = 1310), missed sample (n = 1053), death before sampling (n = 217), and transferals (n = 2). The tested participants were a median age of 6.4 months (interquartile range [IQR]: 1.9–15.0 months) with 48.2% (5001/10 374) aged <6 months and 42.7% (4429/10 374) were female.

Overall, 23.5% (2442/10 374) of the participants tested RSV positive (1413 RSV-A; 996 RSV-B, and 33 RSV-A/B coinfected). Children testing RSV positive were significantly younger (median age 4.1 months; IQR: 1.8–9.3) compared to RSV-negative children (median age 7.5 months, IQR: 1.9–17.0; *P* = .0001). Compared to RSV-negative cases, RSV-positive patients presented more frequently with severe pneumonia (80.7% vs 66.0%; *P*  *<* .001) but less frequently with very severe pneumonia (19.3% vs 34.0%; *P*  *<* .001). This remained significant even after adjusting for sex and age (*P* < .001).

Some specific clinical signs were also more common in RSV-positive children, including chest indrawing (97.9% vs 89.6%), history of cough (96.5% vs 74.7%), history of fever (85.2% vs 75.1%), and crackles (53.8% vs 36.5%) (all *P*  *<* .001) (Table 1). The median length of hospital stay was 4 days in both the RSV-positive and RSV-negative cases. However, the hospital stay lengths differed significantly between the 2 groups (*P <* .001) with RSV-negative patients showing a higher variability of hospital stay (IQR: 2–7 days vs IQR: 3–6 days). Mortality proportion was significantly lower among RSV-positive participants compared to those testing RSV-negative (2.7% vs 10.8%; *P*  *<* .001). Our analysis of the impact of the health care work strikes found that the prolonged strikes in 2020–2021 and 2024 significantly reduced enrollment volumes (*P* < .05) but the shorter industrial actions of <3 weeks had negligible impacts (Supplementary Table 1).

**Table 1.** Baseline Demographic and Clinical Presentation Characteristics of Enrolled Study Participants at KCRH by RSV Status, 2008–2026, and Clinical Discharge Status Outcome

Characteristic	RSV-positive (n = 2442)	RSV-negative (n = 7932)	Total (n = 10 374)	*P* Value
Median age in months (IQR)	4.1 (1.8–9.3)	7.5 (1.9–17.0)	6.4 (1.9–15.0)	<.001
Age categories, n (%)				
0–<6 months	1517 (62.1)	3484 (43.9)	5001 (48.2)	<.001
6–11 months	476 (19.5)	1646 (20.8)	2122 (20.5)	
12–23 months	296 (12.1)	1505 (19.0)	1801 (17.4)	
24–35 months	101 (4.1)	651 (8.2)	752 (7.3)	
36–59 months	52 (2.1)	646 (8.1)	698 (6.7)	
Sex, n (%)				
Male	1409 (57.7)	4536 (57.2)	5945 (57.3)	.654
Symptoms, n (%)				
Fever	2081 (85.2)	5954 (75.1)	8035 (77.5)	<.001
Cough	2357 (96.5)	5927 (74.7)	8284 (79.9)	<.001
Diarrhea	264 (10.8)	1083 (13.7)	1347 (13.0)	<.001
Vomiting	431 (17.7)	1460 (18.4)	1891 (18.2)	.597
Sunken eyes	64 (2.6)	531 (6.7)	595 (5.7)	<.001
Decreased skin turgor	43 (1.8)	396 (5.0)	439 (4.2)	<.001
Chest indrawing	2391 (97.9)	7107 (89.6)	9498 (91.6)	<.001
Crackles	1314 (53.8)	2894 (36.5)	4208 (40.6)	<.001
Wheeze	372 (15.2)	954 (12.0)	1326 (12.8)	<.001
Head nodding	470 (19.3)	920 (11.6)	1390 (13.4)	<.001
Oral candidiasis	9 (0.4)	93 (1.2)	102 (1.0)	<.001
Jaundice	24 (1.0)	194 (2.5)	218 (2.1)	<.001
Pneumonia severity, n (%)				
Severe	1971 (80.7)	5235 (66.0)	7206 (69.5)	<.001
Very severe	471 (19.3)	2697 (34.0)	3168 (30.5)	
Length of hospital stay, median days (IQR)	4 (3–6)	4 (2–7)	4 (3–7)	<.001
In-hospital outcome, n (%)				
Discharged alive	2376 (97.3)	7075 (89.2)	9451 (91.1)	<.001
Died	66 (2.7)	857 (10.8)	923 (8.9)	

Abbreviations: KCRH, Kilifi County Referral Hospital; RSV, respiratory syncytial virus.

### RSV Positivity and Age Distribution Across the Study Phases

When stratified by study phase, RSV positivity declined from 25.3% (1919/7585) in the prepandemic period to 18.3% (235/1282) during the pandemic, remaining similarly low at 19.1% (288/1507) in the postpandemic period (Figure 1*A*).

**Figure 1.**
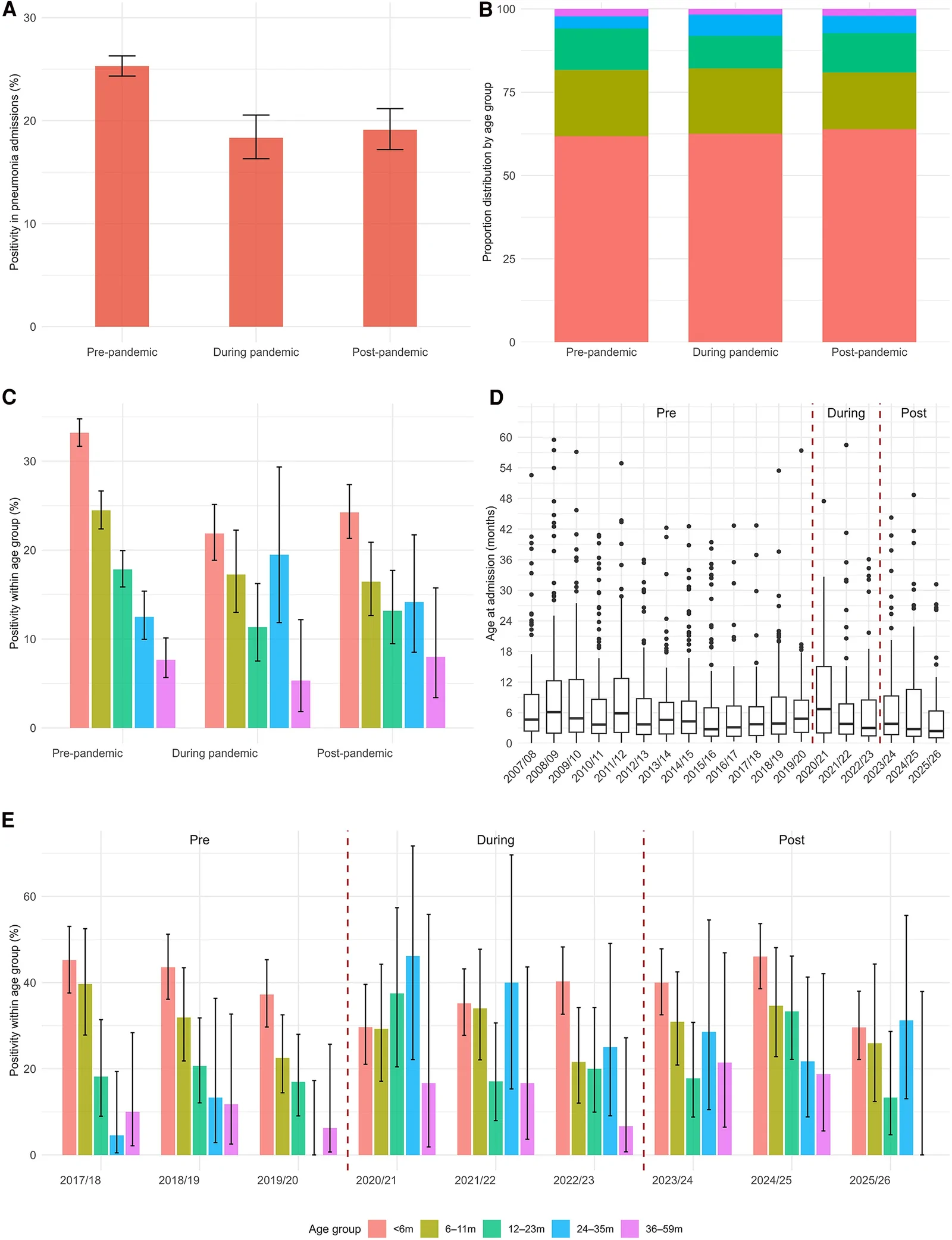
RSV positivity and age distribution across the pandemic phases. (*A*) Changes in overall RSV positivity pre-, during, and postpandemic with 95% CI error bars. (*B*) Distribution of RSV cases by age group across the study period phases: pre-, during, and postpandemic. (*C*) RSV positivity within the different age groups stratified by study phase: pre-, during, and post-COVID-19 pandemic with 95% CI error bars. (*D*) Season-by-season median age distribution box plots of RSV-positive children from the 2007–2008 to 2025–2026 seasons. (*E*) Season-by-season age-stratified RSV positivity within consecutive epidemic seasons (prepandemic: 2017–2018 to 2019–2020; during pandemic: 2020–2021 to 2022–2023; postpandemic: 2023–2024 to 2025–2026) showing 95% CI error bars. CI, confidence interval; RSV, respiratory syncytial virus.

Despite hypotheses suggesting severe RSV disease shift to older age groups, the overall median age for the RSV positive cases in our setting remained statistically similar across the 3 periods: 4.3 months (IQR: 1.8–9.2 months) prepandemic, 3.7 months (IQR: 1.8–9.2 months) during the pandemic, and 3.1 months (IQR: 1.5–9.7 months) postpandemic (*P* = *.108*) (Table 2, Figure 1*D*). Infants aged ≤6 months consistently constituted the largest fraction of RSV-positive children, accounting for approximately 62% of all cases across every phase (pre: 61.8%, during: 62.6%, post: 63.9%; *P* = .508) (Figure 1*B*). Although RSV positivity generally declined with increasing age across all phases, the 24–35 months age group saw a slight, transient increase in positivity (19.5% [11.3–30.1]) during the pandemic period compared to pre- and postpandemic periods (pre: 12.5% [9.9–15.5], post: 14.2% [8.1–22.3]) (Figure 1*C* and Supplementary Table 4). Season-by-season, age-stratified positivity patterns also demonstrated a temporary shift in positivity toward older age categories during the 2020–2021 season (Figure 1*E*).

**Table 2.** Demographic, Clinical, and Virus Subgroup Characteristics of the RSV Cases Pre-, During, and postemergence of COVID-19

Characteristic	Prepandemic (n = 1919)	During pandemic (n = 235)	Postpandemic (n = 288)	Total (n = 2442)	*P* Value
Median age in months (IQR)	4.3 (1.8–9.2)	3.7 (1.8–9.2)	3.1 (1.5–9.7)	4.1 (1.8–9.3)	.108
Age categories, n (%)					
<6 months	1186 (61.8)	147 (62.6)	184 (63.9)	1517 (62.1)	.508
6–11 months	381 (19.9)	46 (19.6)	49 (17.0)	476 (19.5)	
12–23 months	239 (12.5)	23 (9.8)	34 (11.8)	296 (12.1)	
24–35 months	71 (3.7)	15 (6.4)	15 (5.2)	101 (4.1)	
36–59 months	42 (2.2)	4 (1.7)	6 (2.1)	52 (2.1)	
Sex, n (%)					
Male	1107 (57.7)	143 (60.9)	159 (55.2)	1409 (57.7)	.430
Symptoms, n (%)					
Fever	1687 (87.9)	181 (77.0)	213 (74.0)	2081 (85.2)	<.001
Cough	1862 (97.0)	229 (97.5)	266 (92.4)	2357 (96.5)	<.001
Diarrhea	207 (10.8)	31 (13.2)	26 (9.0)	264 (10.8)	.312
Vomiting	341 (17.8)	42 (17.9)	48 (16.7)	431 (17.7)	.897
Sunken eyes	49 (2.6)	10 (4.3)	5 (1.7)	64 (2.6)	.417
Decreased skin turgor	34 (1.8)	5 (2.1)	4 (1.4)	43 (1.8)	.873
Chest indrawing	1882 (98.1)	232 (98.7)	277 (96.2)	2391 (97.9)	.074
Crackles	1024 (53.4)	142 (60.4)	148 (51.4)	1314 (53.8)	.083
Wheeze	280 (14.6)	42 (17.9)	50 (17.4)	372 (15.2)	.453
Head nodding	374 (19.5)	44 (18.7)	52 (18.1)	470 (19.3)	.828
Oral candidiasis	7 (0.4)	1 (0.4)	1 (0.4)	9 (0.4)	.988
Jaundice	20 (1.0)	0 (0.0)	4 (1.4)	24 (1.0)	.236
Pneumonia severity, n (%)					
Severe	1545 (80.5)	200 (85.1)	226 (78.5)	1971 (80.7)	.143
Very severe	374 (19.5)	35 (14.9)	62 (21.5)	471 (19.3)	
Median length of hospital stay, days (IQR)	4 (3.6)	4 (3.6)	4 (3.6)	4 (3.6)	.082
RSV subgroup, n (%)					
RSV-A	1072 (55.9)	154 (65.5)	187 (64.9)	1413 (57.9%)	.002
RSV-B	817 (42.6)	79 (33.6)	100 (34.7)	996 (40.8%)	
RSV-A + B	30 (1.6)	2 (0.9)	1 (0.3)	33 (1.4%)	
In-hospital outcome, n (%)					
Discharged alive	1869 (97.4)	231 (98.3)	276 (95.8)	2376 (97.3)	.191
Died	50 (2.6)	4 (1.7)	12 (4.2)	66 (2.7)	

Abbreviation: RSV, respiratory syncytial virus.

### Shifts in Clinical Presentation and Outcomes

Although core respiratory symptoms remained prevalent, the proportions in specific presentations shifted across the study periods. After adjusting for age, sex, and RSV subgroup, children admitted during the pandemic (aOR = 0.45; 95% CI, 0.32–0.64) and postpandemic (aOR = 0.42; 95% CI, 0.31–0.58) had significantly lower odds of presenting with fever compared with the prepandemic period. Similarly, cough was less common postpandemic (aOR = 0.38; 95% CI, 0.23–0.65) (Figure 2*A*). These associations remained significant after Benjamini–Hochberg adjustment (fever during pandemic: *P* < .001; fever postpandemic: *P* ≤ .001; and cough postpandemic: *P* = .002). No significant differences were observed for crackles, diarrhea, vomiting, sunken eyes, decreased skin turgor, wheeze, head nodding, or oral candidiasis across study periods.

**Figure 2.**
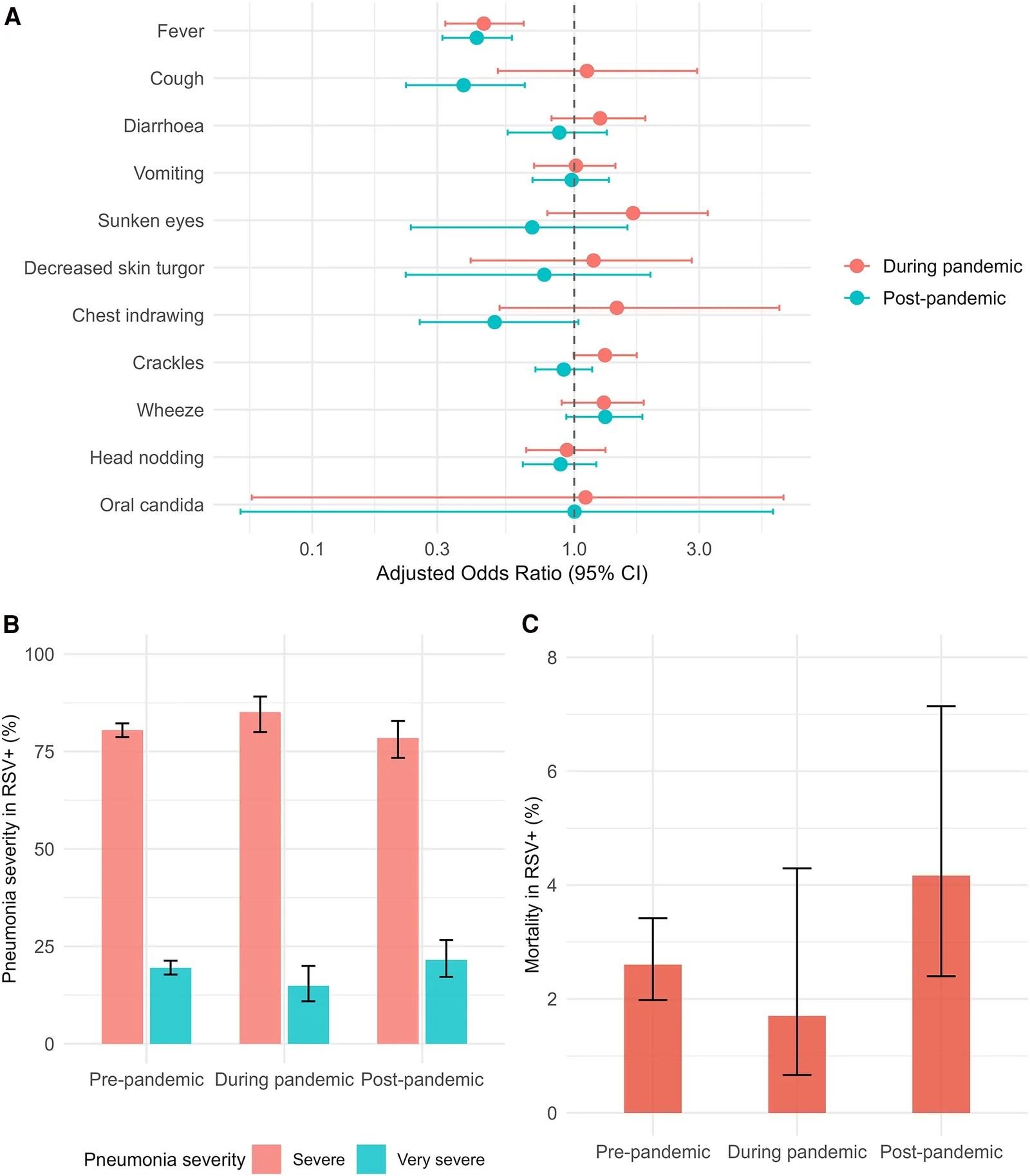
Shifts in RSV clinical presentation, pneumonia severity and inpatient mortality across the pandemic phases. (*A*) A forest plot displaying adjusted odds ratios and 95% CIs for presenting clinical symptoms during the pandemic and postpandemic periods relative to the prepandemic baseline among RSV-positive admissions. Estimates are derived from multivariable logistic regression models adjusted for age, sex, and RSV subgroup. (*B*) Pneumonia severity distribution among the RSV positives across the 3 study period phases. (*C*) In-patient mortality proportions among RSV-positive children across the 3 pandemic phases. CI, confidence interval; RSV, respiratory syncytial virus.

Most children presented with severe pneumonia (pre: 80.5%, during: 85.1%, post: 78.5%) with no significant variation in the proportion experiencing very severe pneumonia across the periods (*P* = *.143*) (Table 2, Figure 2*B*). The median length of hospital stay was 4 days (pre: IQR, 3–6; during: IQR, 3–6; post: IQR, 3–6) across the 3 periods (*P* = *.082*). Overall mortality was 2.6% prepandemic, 1.7% during, and 4.2% postpandemic; this fluctuation was not statistically significant (*P* = .191) (Table 2, Figure 2*C*).

### Seasonality and RSV Subgroup Dynamics

Over the 19 consecutive RSV seasons we observed (13 prepandemic, 3 during, and 3 postpandemic), consistent cyclical patterns were evident (Figure 3*A*). During epidemics, RSV positivity ranged from 26.1% (95% CI, 20.5–32.6) to 63.7% (95% CI, 57.9–69.2) (Supplementary Figure 2*A*). In contrast during the interepidemic period, overall RSV positivity was 4.8% (95% CI, 4.2–5.5).

**Figure 3.**
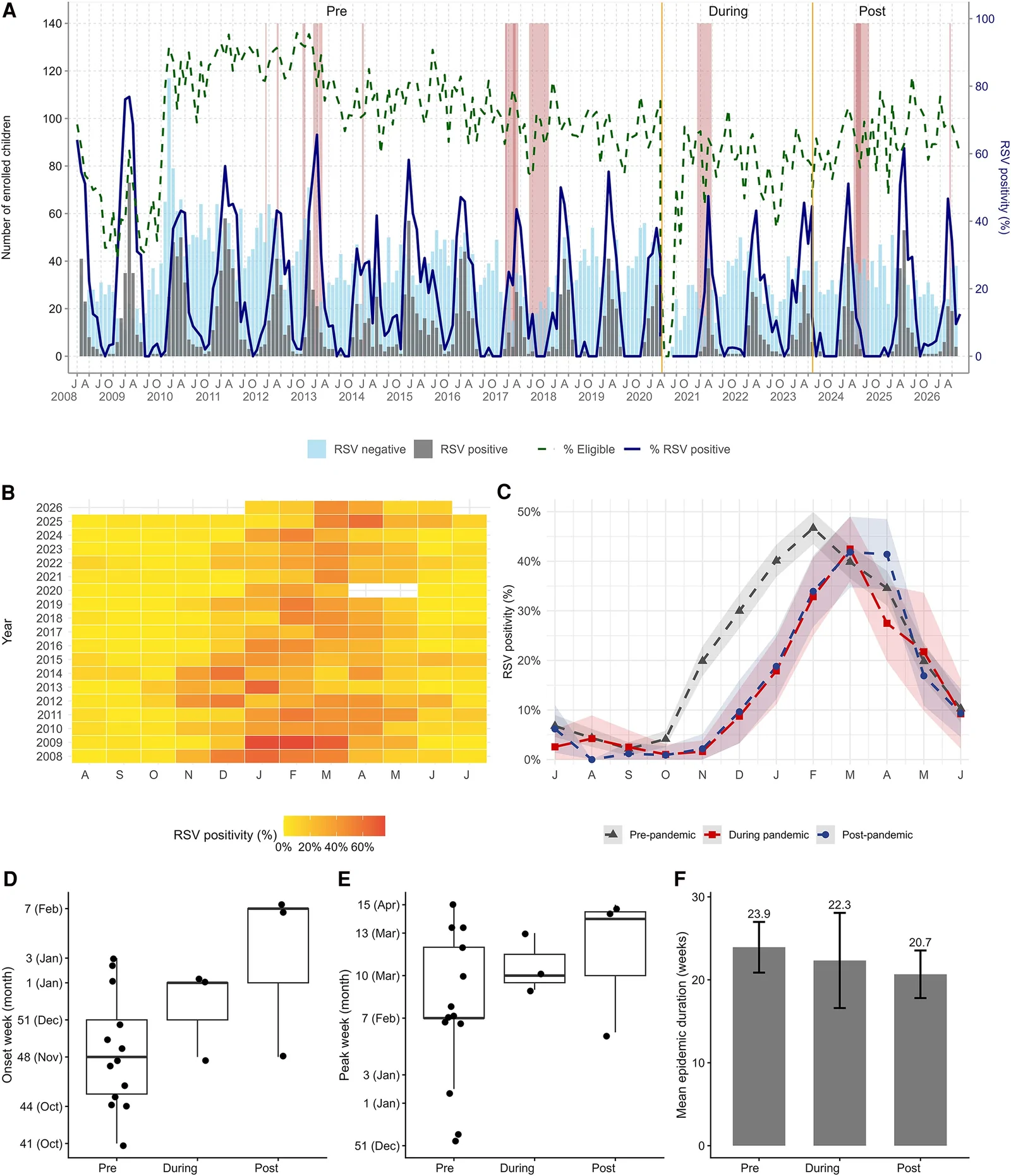
Seasonality and RSV-A and B subgroup dynamics in Kilifi County, coastal Kenya. (*A*) Monthly trends of eligible, and RSV-positive cases among child pneumonia admissions to KCRH. Vertical golden lines delineate pre-, during, and postpandemic periods; pink-shaded bands make nationwide health care worked industrial action periods. (*B*) Heatmap of RSV monthly positivity at KCRH across surveillance years (2008–2026). (*C*) Aggregated mean monthly positivity patterns of RSV in KCRH comparing prepandemic, during pandemic, and postpandemic phases; shaded ribbons indicate 95% confidence intervals. (*D*) Distribution of epidemic onset timing across the 3 phases of the pandemic. (*E*) Distribution of epidemic peak timing across the prepandemic, during pandemic, and postpandemic periods. (*F*) Mean RSV epidemic duration in weeks with error bars representing standard error across the 3 study phases. KCRH, Kilifi County Referral Hospital; RSV, respiratory syncytial virus.

New RSV seasons on the Kenyan coast prepandemic consistently began around October/November/December of 1 year and faded off about April/May/June of the following year after peaking around February/March (Figure 3*B*, Supplementary Table 3) with the exception of the 2016–2017 and 2017–2018 epidemics, which started in January. However, 2 of the 3 pandemic-era epidemics onset of the January of 2 year, all 3 peaked in March of the same year and fading off by May. In the postpandemic period, 2 of the 3 observed epidemics had a February onset, peaked in April and faded off in June/July (Figure 3*C*, Supplementary Table 3). These differences did not attain statistical significance: onset timing (Figure 3*D*, *P* = .146), peak timing (Figure 3*E*, *P* = .360), or epidemic duration (Figure 3*F*, *P* = .459). Sensitivity analyses that reassigned the 2022–2023 epidemic as the postpandemic yielded similar results (onset timing: *P* = .112, peak timing: *P* = .488, epidemic duration: *P* = .378).

Overall, RSV-A predominated the positive cases across the 19 seasons at 57.9% (11/19) (Supplementary Figure 2*C* and *D*). In the prepandemic period, RSV-B predominated in 6 epidemics, whereas RSV-A predominated in 6 with 2009–2010 showing substantial cocirculation of the subgroups (range, 45%–55%). During the pandemic period, RSV-A dominated 2 of 3 epidemics, and 2 of the 3 postpandemic epidemics observed (Supplementary Figure 2). This resulted in the predominance pattern of B, A, AB, A, B, A, A, A, B, B, A, B, B, A, A, B, A, A, B from 2007–2008 to 2025–2026.

## DISCUSSION

We leveraged our long-term RSV surveillance at the Kenyan coast (2008–2026) to investigate RSV epidemiological dynamics pre-, during, and post the COVID-19 pandemic in the region. Similar longitudinal datasets are rare in Africa. A delay of 1–2 months in the onset of local RSV epidemics was observed in 4 of 6 seasons and the prevalence of certain clinical presentations altered but the core demographic and clinical characteristics of RSV cases remained stable, across sexes, age groups, and disease severity categorizations. This is unlike some global reports indicating significant age shifts in RSV severe disease during the pandemic [27, 45–47]. Strikingly, our study confirmed infants aged ≤6 months continued to bear the largest fraction of the RSV disease burden across all the study phases.

Prepandemic, RSV epidemics on the Kenyan Coast typically began around October/November of 1 year (with few exceptions; Supplementary Table 3) and ended around April/May, with a peak in the months of January/February/March [14, 35]. However, a delayed onset of the local 2020–2021 RSV epidemic is reported here (started in January 2021), which aligns with global reports indicating the unprecedented disruption of seasonal transmission of endemic respiratory viruses majorly due to the COVID-19 NPIs [16]. However, notably, most of the subsequent local epidemics during and postpandemic continued to record a delayed onset compared to the historical patterns. Although this delay may be linked to the NPIs that were in place in the early COVID-19 pandemic years, other factors such as the RSV-predominant subgroup, new RSV global interventions, and virus–virus interaction (also called “virus interference”) may also contribute to these altered seasonal patterns [48, 49]. Notably recent SARS-CoV-2 epidemic waves in coastal Kenya have been onsetting around November of 1 year, peaking in December/January and fading by February of the following year [50] (Supplementary Figure 3).

When the COVID-19 NPIs were relaxed, several high-income countries reported interseasonal transmission and surges of severe RSV in older pediatrics populations (e.g., in Australia and France) [51, 52] (Supplementary Table 6). In France, compared to previous outbreaks, the 2020–2021 epidemic involved more children aged 6 to 11 months (25.8% vs 13.1%, *P* < .0001), but few infants aged <6 months (41.3% vs 56.6% previously, *P* < .0001) [52]. In China, after the lifting of NPIs, delayed RSV outbreaks occurred in the 2022–2023 season with unusual seasonality and infections increasing markedly in the 3- to 6-year age group [53]. This phenomenon has been attributed to an “immunity debt” acquired during the preceding months of reduced RSV transmission [54–56]. In the United States, both delayed seasonality and [17] hospitalization of older children during 2022–2023 (median 11.3 months) versus prepandemic (6.8 months) were observed [57]. However, it has been also hypothesized that increased in positivity in older children may be also related to the changes in testing practices [24].

In our coastal Kenya surveillance, like reported globally [58, 59], we observed a decrease of RSV positivity both during (18.3%) and postpandemic (19.1%) compared to the prepandemic period (25.3%). This decrease was also evident in <6-month olds and 6- to 11-month olds. Unexpectedly, the change was accompanied with a slight overall decrease in the median age for children admitted with RSV from the prepandemic (4.3) to during (3.7) and the postpandemic period (3.1) (*P* = .108). We only observed a slight transient increase in RSV positivity in the 24- to 35-month olds (12.5% vs 19.5% vs 14.2%, *P* = .233). Notably, however, a season-by-season analysis revealed that the 2020–2021 epidemic exhibit a higher median age of 6.7 (IQR: 2.0–15.1) months, presenting a transient, short-lived “immunity debt” signal like that reported in higher-income settings.

Notably, overall, ∼62% of RSV-associated admissions in our setting consistently occurred in infants aged ≤6 months. The lack of a statistically significant age shift mirrors observations reported in Croatia [60] and Greece [59], suggesting that the “immunity debt” phenomenon may not have displaced the primary disease burden away from young infants. Potential explanations for this include: (1) NPIs were not exactly same worldwide, (2) testing practices for RSV varied by country, and (3) inherent differences in RSV epidemiology in different transmission settings [61]. Furthermore, there are differences in how “during” and “postpandemic” phases are defined across studies.

The 2 RSV groups typically cocirculate, alternating predominance in consecutive epidemics [62, 63]. Our study found similar distribution of RSV-A and RSV-B cases in severe and very severe pneumonia. Overall, pneumonia severity and mortality among RSV-positive cases did not differ significantly pre-, during, and postpandemic, although we noted significant shifts in the prevalence of some symptoms (e.g., fever). Overall, among children admitted with severe or very severe pneumonia, being RSV negative predicted a worse outcome compared to being RSV positive.

Our study has some limitations. First, the number of enrolled participants considerably fluctuated across the study period, influenced by consenting rates, health worker industrial action and the arrival of both the 2009 swine flu pandemic [64, 65] and COVID-19 pandemic, which may have modified the study population’s health-seeking behavior. Second, our analysis did not interrogate patterns for other respiratory viruses because this has potential to influence the observed RSV epidemic patterns through virus–virus interference [48, 49]. Third, our study relied on data from a single site and the findings may not be well representative of the patterns across the entire Kenya or other African countries. Fourth, we updated the RSV-B PCR assay twice during the surveillance due to observed primer/probe mismatches in contemporaneous strains. This may have impacted the subgroup distribution in some of the epidemics when the RSV-B assay may have missed. However, substantial to almost perfect diagnostic agreement between RT-PCR and our continuous antigen-detection (Cohen's Kappa > 0.70 across seasons; Supplementary Table 5 and Supplementary Figure 4). Finally, this analysis did not examine the circulating genotype distribution within the RSV subgroups which may have an influence on the epidemic patterns.

In conclusion, despite the transmission disruptions caused by the COVID-19 pandemic and its associated NPIs globally, RSV exhibited a consistent regular epidemic pattern on the Kenya Coast, albeit with multiple delayed epidemic onsets during and in the postpandemic era. Infants aged <6 months remain the most vulnerable population. Minor changes in RSV disease clinical presentation occurred across the different study phases following COVID-19 pandemic. Our study underscores the importance of sustained long-term studies to guide optimal timing and deployment of emerging RSV interventions such as the long-acting monoclonal antibodies and maternal vaccination to protect the unborn child in their first few months of life.

## Supplementary Material

ofag600_Supplementary_Data

## Supplementary Data

Supplementary materials are available at *Open Forum Infectious Diseases* online. Consisting of data provided by the authors to benefit the reader, the posted materials are not copyedited and are the sole responsibility of the authors, so questions or comments should be addressed to the corresponding author.
