# Supplementary material for: Delayed Seasonality but Consistent Age Burden: RSV Epidemiology Pre–, During, and Post–COVID-19 Pandemic on the Kenyan Coast (2008–2026)
Source: Open Forum Infect Dis. 2026 Sep 22;13(10):ofag600. doi: 10.1093/ofid/ofag600 (PMC13660703; doi:10.1093/ofid/ofag600)
Supplement: Supplementary file 1 [file ofag600_supplementary_data.docx]

## **Delayed seasonality but consistent age burden: RSV epidemiology pre-, during and post-COVID-19 pandemic on the Kenyan Coast (2008-2026)**

# Supplementary Tables

**Supplementary Table 1**: Timing and duration of nationwide healthcare worker strikes that occurred between January 2008 to June 2026 with comparison of surveillance metrics from similar non-strike calendar periods

| Industrial action event | Start date | End date | Duration (days) | Samples collected during event (n) | RSV positive during event (n) | RSV positivity during event, % (95% CI) | Mean samples in same period, other years | Mean RSV positive in same period, other years | Mean RSV positivity in same period, other years, % (95% CI) | P-value^¶^ |
| --- | --- | --- | --- | --- | --- | --- | --- | --- | --- | --- |
| Doctors' strike | 05-Dec-11 | 13-Dec-11 | 9 | 12 | 0 | 0.0 (0-26.5) | 14.5 | 3.6 | 25.0 (19.6-31.1) | 0.047 |
| Nurses' strike | 01-Mar-12 | 15-Mar-12 | 15 | 36 | 17 | 47.2 (30.4-64.5) | 33.2 | 13.9 | 41.8 (37.8-45.9) | 0.523 |
| Doctors' strike | 13-Sep-12 | 04-Oct-12 | 22 | 39 | 1 | 2.6 (0.1-13.5) | 27.2 | 0.5 | 1.9 (0.9-3.7) | 0.790 |
| Nurses' strike | 03-Dec-12 | 13-Jan-13 | 42 | 59 | 34 | 57.6 (44.1-70.4) | 71.0 | 18.5 | 26.1 (23.5-28.7) | <0.001 |
| Nurses' strike | 16-Jan-13 | 11-Feb-13 | 27 | 21 | 13 | 61.9 (38.4-81.9) | 56.4 | 22.4 | 39.8 (36.8-42.9) | 0.041 |
| Doctors' and nurses' strike | 10-Dec-13 | 23-Dec-13 | 14 | 7 | 2 | 28.6 (3.7-71.0) | 20.6 | 5.2 | 25.4 (20.9-30.2) | 0.847 |
| Nurses' strike | 05-Dec-16 | 15-Dec-16 | 11 | 0 | 0 | NA**^‡^** | 17.9 | 4.4 | 24.7 (19.9-29.9) | NA |
| Doctors' strike | 05-Dec-16 | 15-Mar-17 | 101 | 106 | 28 | 26.4 (18.3-35.9) | 204.1 | 74.4 | 36.4 (34.8-38.1) | 0.034 |
| Nurses' strike | 02-Feb-17 | 22-Feb-17 | 21 | 13 | 3 | 23.1 (5.0-53.8) | 49.3 | 21.8 | 44.3 (41.0-47.6) | 0.127 |
| Nurses' strike | 05-Jun-17 | 02-Nov-17 | 151 | 95 | 3 | 3.2 (0.7-9.0) | 178.5 | 8.6 | 4.8 (4.1-5.6) | 0.460 |
| Healthcare workers’ strike | 07-Dec-20 | 26-Mar-21 | 110 | 122 | 34 | 27.9 (20.1-36.7) | 219.7 | 82.2 | 37.4 (35.8-39.0) | 0.032 |
| Doctors' strike | 13-Mar-24 | 08-May-24 | 57 | 99 | 13 | 13.1 (7.2-21.4) | 101.7 | 37.6 | 36.9 (34.7-39.2) | <0.001 |
| Clinical officers strike | 01-Apr-24 | 08-Jul-24 | 99 | 127 | 8 | 6.3 (2.8-12.0) | 130.2 | 30.8 | 23.6 (21.9-25.4) | <0.001 |
| Nurses' strike | 12-Mar-26 | 23-Mar-26 | 12 | 19 | 10 | 52.6 (28.9-75.6) | 27.1 | 10.7 | 39.3 (35.0-43.8) | 0.246 |

CI: confidence interval, NA, not applicable, RSV, respiratory syncytial virus; ^‡^Not applicable due to zero patient enrollment/sampling during the brief 11-day strike; ¶P-values were calculated using two-sample test of proportions comparing the proportion of RSV-positive samples during the strike period with the pooled average across the same calendar dates in non-strike years.

**Supplementary Table 2**: Real-time RT-PCR diagnostic primers and probes used for RSV detection and subgroup differentiation at KEMRI-Wellcome Trust Research Programme (2008-2026)

| Subgroup | Period of application | Oligonucleotide type | Sequence and modification (5'-3') | Target gene | Reference |
| --- | --- | --- | --- | --- | --- |
| RSV-A | Jan 2008 - Jun 2026 | Forward Primer | AGATCAACTTCTGTCATCCAGCAA | N | (1) |
|  |  | Reverse Primer | TTCTGCACATCATAATTAGGAG |  |  |
|  |  | Probe | 6FAM-CACCATCCAACGGAGCACAGGAGAT-BHQ |  |  |
| RSV-B | Jan 2008 - Jun 2016 | Forward Primer | AAGATGCAAATCATAAATTCACAGGA | N | (1) |
|  |  | Reverse Primer | TGATATCCAGCATCTTTAAGTA |  |  |
|  |  | Probe | VIC-TTTCCCTTCCTAACCTGGACATA-TAMRA |  |  |
|  | Jul 2016 - Dec 2024 | Forward Primer | GCATCATTGCTGTCATTAACTCAATT |  | (2) |
|  |  | Reverse Primer | GGTGTACCTCTRTACTCTCCCATTATG |  |  |
|  |  | Probe | VIC-TCAAGTGTGGTCYTAGGYAATGCAGC-TAMRA |  |  |
|  | Jan 2025 - Jun 2026 | Forward Primer | AATACAGAAAAATCTAACCAACTTTACA | L | (3) |
|  |  | Reverse Primer | CCAAGGAAGCATGCAATAAA | L |  |
|  |  | Probe | VIC-GACATCTTTAGTAAGGAATAGTG-TAMRA | L |  |

Note: Samples tested prior to the introduction of assay modification were not retrospectively retested; original subtype classifications and cycle threshold (Ct) were retained for analysis; **Target gene:** *N*, nucleocapsid protein gene; *L*, large polymerase protein gene.

**Supplementary Table 3:** Season-by-season epidemiological characteristics, timing (onset, peak, offset), duration, testing volumes, positivity rates and predominant subgroup across 19 consecutive RSV seasons at Kilifi County Referral Hospital (2007/08-2025/26).

| Season | Study phase | Epidemic onset (Week, Month)^*^ | Epidemic peak (Week, Month) | Epidemic offset (Week, Month) | Duration (Weeks) | Tested (N) | RSV positive (n) | RSV positivity (%) (95% CI) | Inter-epidemic interval (Weeks) | Predominant subgroup^‡^ |
| --- | --- | --- | --- | --- | --- | --- | --- | --- | --- | --- |
| 2007/08 | Pre-pandemic | 46 (Nov) | 2 (Jan) | 18 (Apr) | 18 | 259 | 138 | 53.3 | 33 | RSV-B |
| 2008/09 | Pre-pandemic | 46 (Nov) | 7 (Feb) | 17 (Apr) | 24 | 273 | 174 | 63.7 | 32 | RSV-A |
| 2009/10 | Pre-pandemic | 49 (Dec) | 10 (Mar) | 17 (Apr) | 22 | 559 | 195 | 34.9 | 28 | RSV-AB |
| 2010/11 | Pre-pandemic | 45 (Nov) | 13 (Mar) | 22 (May) | 30 | 570 | 231 | 40.5 | 26 | RSV-A |
| 2011/12 | Pre-pandemic | 48 (Nov) | 13 (Mar) | 22 (May) | 27 | 419 | 126 | 30.1 | 22 | RSV-B |
| 2012/13 | Pre-pandemic | 44 (Oct) | 51 (Dec) | 16 (Apr) | 25 | 303 | 119 | 39.3 | 25 | RSV-A |
| 2013/14 | Pre-pandemic | 41 (Oct) | 15 (Apr) | 21 (May) | 33 | 386 | 101 | 26.2 | 23 | RSV-A |
| 2014/15 | Pre-pandemic | 44 (Oct) | 52 (Dec) | 22 (May) | 31 | 427 | 162 | 37.9 | 27 | RSV-A |
| 2015/16 | Pre-pandemic | 49 (Dec) | 8 (Feb) | 17 (Apr) | 22 | 356 | 137 | 38.5 | 37 | RSV-B |
| 2016/17 | Pre-pandemic | 2 (Jan) | 12 (Mar) | 22 (May) | 21 | 209 | 70 | 33.5 | 33 | RSV-B |
| 2017/18 | Pre-pandemic | 3 (Jan) | 7 (Feb) | 21 (May) | 19 | 301 | 105 | 34.9 | 30 | RSV-A |
| 2018/19 | Pre-pandemic | 51 (Dec) | 7 (Feb) | 20 (May) | 22 | 327 | 110 | 33.6 | 27 | RSV-B |
| 2019/20 | Pre-pandemic | 47 (Nov) | 7 (Feb) | 11 (Mar) | 17 | 313 | 83 | 26.5 | 44 | RSV-B |
| 2020/21 | During pandemic | 1 (Jan) | 9 (Mar) | 21 (May) | 21 | 175 | 55 | 31.4 | 28 | RSV-A |
| 2021/22 | During pandemic | 1 (Jan) | 10 (Mar) | 21 (May) | 21 | 258 | 81 | 31.4 | 27 | RSV-A |
| 2022/23 | During pandemic | 48 (Nov) | 13 (Mar) | 20 (May) | 25 | 271 | 84 | 31.0 | 28 | RSV-B |
| 2023/24 | Post-pandemic | 48 (Nov) | 6 (Feb) | 17 (Apr) | 22 | 296 | 98 | 33.1 | 44 | RSV-A |
| 2024/25 | Post-pandemic | 7 (Feb) | 15 (Apr) | 28 (Jul) | 22 | 313 | 121 | 38.7 | 31 | RSV-A |
| 2025/26 | Post-pandemic | 7 (Feb) | 14 (Apr) | 26 (Jun) | 20 | 203 | 53 | 26.1 | - | RSV-B |

*Epidemic onset was defined as the first week of at least two consecutive weeks with 10% RSV positivity or ≥2 laboratory-confirmed cases, provided the epidemic lasted for a minimum of 4 consecutive weeks. Epidemic offset was defined as the last week meeting these criteria before dropping below threshold levels; ^‡^ Predominant subgroup was defined as the subgroup accounting for ≥55% of all subtyped RSV cases during that specific epidemic season. RSV-AB denotes co-dominance/substantial co-circulation where neither subgroup met the ≥55% threshold.

**Supplementary Table 4**: Age-stratified RSV positivity across the pre-, during and post-pandemic study periods.

| Age category | Pre-pandemic tested (N) | Pre-pandemic RSV positive (n) | Pre-pandemic RSV positivity (% (95% CI)) | During pandemic tested (N) | During pandemic RSV positive (n) | During pandemic RSV positivity (% (95% CI)) | Post-pandemic tested (N) | Post-pandemic RSV positive (n) | Post-pandemic RSV positivity % (95% CI) | p-value |
| --- | --- | --- | --- | --- | --- | --- | --- | --- | --- | --- |
| <6months | 3571 | 1186 | 33.2 (31.7, 34.8) | 669 | 147 | 22.0 (18.9, 25.3) | 761 | 184 | 24.2 (21.2, 27.4) | <0.001 |
| 6–11months | 1557 | 381 | 24.5 (22.4, 26.7) | 257 | 46 | 17.9 (13.4, 23.1) | 308 | 49 | 15.9 (12.0, 20.5) | <0.001 |
| 12–23months | 1340 | 239 | 17.8 (15.8, 20.0) | 204 | 23 | 11.3 (7.3, 16.4) | 257 | 34 | 13.2 (9.3, 18.0) | 0.019 |
| 24–35months | 569 | 71 | 12.5 (9.9, 15.5) | 77 | 15 | 19.5 (11.3, 30.1) | 106 | 15 | 14.2 (8.1, 22.3) | 0.233 |
| 36–59months | 548 | 42 | 7.7 (5.6, 10.2) | 75 | 4 | 5.3 (1.5, 13.1) | 75 | 6 | 8.0 (3.0, 16.6) | 0.754 |
| Overall (0-59 months) | 7585 | 1919 | 25.3% (24.3–26.3) | 1,282 | 235 | 18.3% (16.2–20.6) | 1,507 | 288 | 19.1% (17.2–21.2) | <0.001 |

CI: Confidence interval; RSV: respiratory syncytial virus; N: total children tested for RSV in the age stratum; n: number of RSV positive cases

**Supplementary Table 5**: Season-by-season comparison of RSV positivity by RT-PCR and IFAT over the study epidemics.

| Epidemic season | Surveillance phase | Total IFAT tested (N) | IFAT positive, n (%) | Total RT-PCR tested (N) | RT-PCR positive, n (%) | Predominant subgroup^‡^ | Cohen’s kappa-value | Comment |
| --- | --- | --- | --- | --- | --- | --- | --- | --- |
| 2007/08 | Pre-pandemic | 258 | 107 (41.5) | 259 | 138 (53.3) | RSV-B | 0.717 | Only a fraction of the epidemic is captured as our RT-PCR surveillance reported here started January 1, 2008 |
| 2008/09 | Pre-pandemic | 273 | 151 (55.3) | 273 | 174 (63.7) | RSV-A | 0.796 |  |
| 2009/10 | Pre-pandemic | 559 | 193 (34.5) | 559 | 195 (34.9) | RSV-AB | 0.747 |  |
| 2010/11 | Pre-pandemic | 570 | 222 (38.9) | 570 | 231 (40.5) | RSV-A | 0.901 |  |
| 2011/12 | Pre-pandemic | 418 | 119 (28.5) | 419 | 126 (30.1) | RSV-B | 0.913 | Season with doctors' and nurses' strike |
| 2012/13 | Pre-pandemic | 303 | 123 (40.6) | 303 | 119 (39.3) | RSV-A | 0.916 | Season with doctors' and nurses' strike |
| 2013/14 | Pre-pandemic | 385 | 106 (27.5) | 386 | 101 (26.2) | RSV-A | 0.914 | Season with doctors' and nurses' strike |
| 2014/15 | Pre-pandemic | 427 | 160 (37.5) | 427 | 162 (37.9) | RSV-A | 0.900 |  |
| 2015/16 | Pre-pandemic | 354 | 153 (43.2) | 356 | 137 (38.5) | RSV-B | 0.819 |  |
| 2016/17 | Pre-pandemic | 208 | 78 (37.5) | 209 | 70 (33.5) | RSV-B | 0.758 | Season with RSV-B assay update and doctors' and nurses' strike |
| 2017/18 | Pre-pandemic | 301 | 138 (45.8) | 301 | 105 (34.9) | RSV-A | 0.748 | Season with nurses' strike |
| 2018/19 | Pre-pandemic | 326 | 112 (34.4) | 327 | 110 (33.6) | RSV-B | 0.918 |  |
| 2019/20 | Pre-pandemic | 311 | 87 (28.0) | 313 | 83 (26.5) | RSV-B | 0.935 |  |
| 2020/21 | During-pandemic | 173 | 70 (40.5) | 175 | 55 (31.4) | RSV-A | 0.798 | Season with healthcare workers' strike |
| 2021/22 | During-pandemic | 235 | 84 (35.7) | 258 | 81 (31.4) | RSV-A | 0.808 |  |
| 2022/23 | During-pandemic | 228 | 42 (18.4) | 271 | 84 (31.0) | RSV-B | 0.572 | A few samples missed the IFAT test due to kit supply issue |
| 2023/24 | Post-pandemic | 295 | 102 (34.6) | 296 | 98 (33.1) | RSV-A | 0.834 | Season with doctors' strike |
| 2024/25 | Post-pandemic | 312 | 102 (32.7) | 313 | 121 (38.7) | RSV-A | 0.771 | Season RSV-B assay update |
| 2025/26 | Post-pandemic | 203 | 46 (22.7) | 203 | 53 (26.1) | RSV-B | 0.773 | Season with nurses' strike |

RSV, respiratory syncytial virus; RT-PCR, reverse transcription real-time polymerase chain reaction; Note: Cohen’s kappa measures diagnostic agreement beyond chance between IFAT and real-time RT-PCR: values >0.80 denote almost perfect agreement; 0.61–0.80 substantial agreement; and 0.41–0.60 moderate agreement. ^‡^ Predominant subgroup was defined as the subgroup accounting for ≥55% of all subtyped RSV cases during that specific epidemic season. RSV-AB denotes co-dominance/substantial co-circulation where neither subgroup met the ≥55% threshold.

**Supplementary Table 6**: Global evidence on changes in RSV seasonality and pediatric age distribution pre-, during and post COVID-19 pandemic

| Study/Reference | Country/Region | Study setting | Study period | Direction of seasonal change | Seasonality summary | Direction of age shift | Age distribution summary |
| --- | --- | --- | --- | --- | --- | --- | --- |
| Hu et al (2026) (4) | China | Inpatient and outpatient | Jan 2022 to Dec 2025 | Post-pandemic variation | Year-to-year variation in post-pandemic activity; study period was largely post-pandemic and does not establish a pre- to post-pandemic shift. | No consistent shift | RSV remained concentrated in younger children. Median age varied from 1.4 to 2.6 years across 2022-2025 indicating modest year-to-year variation rather than a sustained shift toward older ages. |
| Rave et al. (2026) (5) | Netherlands | Inpatient | May 2021 to Apr 2024 | Off-season to normal | An unusual summer RSV outbreak occurred in 2021 was followed by return toward typical winter seasonality with post-pandemic peak occurring ~ 1 month later than pre-pandemic timing. | Older age during pandemic, then return toward pre pandemic | Median age at admission increased from 2.2 months pre-pandemic to 4.9 months during the 2021 summer outbreak, subsequently returning to 2.7 months by 2023/24. |
| Fujii et al. (2026) (6) | Japan | Inpatient | 2018 to 2024 | Earlier | Epidemic peaks shifted from August in pre-pandemic seasons to March in 2023–2024, indicating altered seasonal timing. | Older age followed by gradual return toward pre-pandemic | The proportion of hospitalized patients aged ≥2 years rose to ~ 30% in 2021–2022 (vs <20% pre-pandemic), declining to 21.2% in 2024. |
| Chang et al. (2026) (7) | Taiwan | Inpatient | Jan 2015 to Dec 2023 | Delayed / concentrated season | Shifted from year-round circulation with July–September peaks to a more concentrated September–December epidemic after 2020. | Older age | Proportion of cases among infants<1 year decreased from 56.5% to 24.1%, while the proportion among children >2 years increased significantly. |
| Park et al (2025) (8) | South Korea | Inpatient + outpatient | Sep 2017 to Aug 2024 | Delayed / prolonged | Post-pandemic RSV epidemics were delayed and prolonged, with atypical epidemic timing and multiple peaks in some seasons. | Older age | Proportion of hospitalizations among children aged 1-6 years increased from 48% pre-pandemic to 57% in 2021/22 and 61% in 2022/23. |
| Philip et al. (2026) (9) | Ireland | National surveillance | 2012 to 2024 | Earlier | Onset and peak occurred ~2 months earlier post-pandemic than pre-pandemic, with higher and more concentrated epidemic peaks. | Older preschool age | Incidence increased substantially among children aged 1-4 years post-pandemic; (more than four-fold higher in 2023/24 than pre-COVID levels. |
| Zhou et al. (2026) (10) | China | Pediatric respiratory surveillance | Sep 2017 to Aug 2024 | Delayed / atypical to partial normalization | Delayed, atypical outbreak occurred following NPI relaxation (unusual April peak in 2022/23), followed by partial normalization in 2023/24. | Older preschool age | Infections increased markedly among children aged 3-6 years in 2023, consistent with redistribution toward older preschool children. |
| Masoom et al. (2025) (11) | USA | Pediatric emergency departments | 2018-2023 | Disrupted / atypical | Seasonality deviated substantially from established historical timing following the pandemic onset. | Older age | RSV-positive patients in seasons after the COVID-19 onset were significantly older than those in seasons (median: 0.83 years vs. 0.56 years; p < 0.001). |
| Perramon-Malavez et al. (2024) (12) | Spain | Tertiary pediatric hospital | Sept 2014 to Jan 2024 | Return toward pre-pandemic seasonality | 2023/2024 seasonal pattern resembled pre-pandemic pattern despite substantial community transmission. | Older age | 12-35 months carried the main RSV community burden; relative risk among infants declined substantially. |
| Perramon-Malavez et al. (2023) (13) | Spain | Tertiary pediatric hospital/PICU | 2016-2023 | Delayed to earlier onset | RSV activity declined during pandemic; 2021 epidemic occurred in May–July; 2022/2023 epidemic began 4-6 weeks earlier than usual. | Predominantly infants | ≤3-month remained the predominant group, although older children contributed substantially during disrupted seasons. |
| Winthrop et al. (2024) (14) | USA | Inpatient | Jul 2017 to Jun 2023 | Post-pandemic resurgence | Increase in RSV hospital presentations and hospitalizations in 2022/2023. | Older age | Median age increased from 6.8 to 11.3 months. |
| Mrcela et al. (2022) (15) | Croatia | Inpatient | Jan 2018 to Jun 2022 | Disrupted | Suppressed winter 2020/2021 epidemic followed by late spring/summer 2021 resurgence; hospitalization rates subsequently returned to pre-pandemic levels. | No major shift | RSV hospitalization remained highest among infants <6 months, post-resurgence age distribution was broadly similar to pre-pandemic. |
| Berikopoulou et al. (2024) (16) | Greece | Tertiary pediatric hospital | Jan 2018 to May 2023 | Earlier peak | Peak shifted from January-March pre-pandemic to October-December post-pandemic. | Older age | Mean age increased from 5.9 months pre-pandemic to 16.7 months post-pandemic. |

# Supplementary Figures

**Supplementary Figure 1:** Flowgram of participant recruitment, RSV testing and subgroup distribution across the 3 study periods. Schematic flowchart showing the number of eligible paediatric admissions presenting with WHO syndromic severe or very severe pneumonia in Kilifi County Referral Hospital between January 2008 and June 2026, those tested and not tested for RSV. The number of RSV-positive cases by period and RSV subgroup.

**Supplementary Figure 2: Longitudinal RSV positivity patterns and subgroup dynamics across 19 consecutive surveillance seasons. A)** RSV positivity per epidemic season across the 19 consecutive surveillance seasons (2007/08-2025/26). Error bars denote 95% confidence intervals **B)** Distribution of real-time RT-PCR Ct value among positives stratified by RSV subgroup. Center lines indicate medians; box hinges indicate the 25th and 75th percentiles (IQR); whiskers extend to 1.5× IQR; individual points represent outlying values **C)** Overall RSV positivity by subgroup across the three study phases with error bars indicating 95% CI. **D)** Proportional distribution of RSV-A and RSV-B positivity across each epidemic season and inter-epidemic intervals.

**Supplementary Figure 3:** Longitudinal trends in SARS-CoV-2 monthly testing and monthly positivity pattern among pediatric pneumonia admissions in Kilifi County Referral Hospital (2020-2026).

**Supplementary Figure 4:** Longitudinal comparison of monthly RSV detections by real-time RT-PCR and immunoflourescence antibody testing (IFAT) at Kilifi Country Referral Hospital over the study period (2008-2026). Vertical dashed lines indicate changes in RSV-B diagnostic assay in July 2016 and January 2025.

References:

1.	Gunson RN, Collins TC, Carman WF. Real-time RT-PCR detection of 12 respiratory viral infections in four triplex reactions. J Clin Virol. 2005;33(4):341-4.

2.	Kamau E, Agoti CN, Lewa CS, Oketch J, Owor BE, Otieno GP, et al. Recent sequence variation in probe binding site affected detection of respiratory syncytial virus group B by real-time RT-PCR. J Clin Virol. 2017;88:21-5.

3.	Williams T, Jackson S, Barr I, Bi S, Bhiman J, Ellis J, et al. Results from the second WHO external quality assessment for the molecular detection of respiratory syncytial virus, 2019-2020. Influenza Other Respir Viruses. 2023;17(1):e13073.

4.	Hu H. Post‑pandemic shifts in the age distribution of RSV, influenza A, and rhinovirus infections: A four‑year pediatric surveillance study. BMC Public Health. 2026;26(1):2292.

5.	Rave N, de Bruijne MEM, Stellato RK, Bannier M, Weinberger DM, Löwensteyn YN, et al. Normalization of Seasonality and Age Distribution of Pediatric RSV Infection Following the Pandemic Disruption in the Netherlands. Open Forum Infect Dis. 2026;13(1):ofaf759.

6.	Fujii M, Ohnishi T, Moriwaki K, Kunikata T. Ongoing Shift Toward an Earlier RSV Season in Children: The Case of Saitama After the COVID-19 Pandemic. Japanese Journal of Infectious Diseases. 2026;79(4):168-71.

7.	Chang TH, Hu YL, Chen WC, Fang CT, Ho SY, Lee TF, et al. Age redistribution and altered RSV seasonality in Taiwan: A multicenter analysis from 2015 to 2023. Int J Infect Dis. 2026;162:108181.

8.	Park M, Choi WS, Cowling BJ. Shifts in Influenza and Respiratory Syncytial Virus Infection Patterns in Korea After the COVID-19 Pandemic Resulting From Immunity Debt: Retrospective Observational Study. JMIR Public Health Surveill. 2025;11:e68058.

9.	Philip RK, Mangroo K, Gendy N, Purtill H, Kelly E, Domegan L, et al. Respiratory syncytial virus (RSV) notifications and trends in the transmission cycles from infants and young children to older adults in Ireland: an analysis of incidence shifts over a decade. BMJ Open. 2026;16(1):e107050.

10.	Zhou Y, Xia JB, Luo M, Zhang WX, Zhang SS, Ao L, et al. Shifts in epidemic patterns and age dynamics of RSV, IFV, and SARS-CoV-2 in Chinese children following COVID-19 non-pharmaceutical intervention relaxation, 2017 - 2024. Eur J Clin Microbiol Infect Dis. 2026;45(1):207-16.

11.	Masoom SI, Aloisio G, Camp EA, Dunn JJ, Meskill SD. Characterizing respiratory syncytial virus (RSV) infections before and during the COVID-19 pandemic. Am J Emerg Med. 2025;87:108-12.

12.	Perramon-Malavez A, de Rioja VL, Coma E, Hermosilla E, Fina F, Martínez-Marcos M, et al. Introduction of nirsevimab in Catalonia, Spain: description of the incidence of bronchiolitis and respiratory syncytial virus in the 2023/2024 season. Eur J Pediatr. 2024;183(12):5181-9.

13.	Perramon-Malavez A, López de Rioja V, Creus-Costa A, Andrés C, Montañola-Sales C, Vila J, et al. Unraveling the effects of the COVID-19 pandemic on the hospital burden of respiratory syncytial virus. Pediatr Pulmonol. 2023;58(10):2979-82.

14.	Winthrop ZA, Perez JM, Staffa SJ, McManus ML, Duvall MG. Pediatric Respiratory Syncytial Virus Hospitalizations and Respiratory Support After the COVID-19 Pandemic. JAMA Netw Open. 2024;7(6):e2416852.

15.	Mrcela D, Markic J, Zhao C, Viskovic DV, Milic P, Copac R, et al. Changes following the Onset of the COVID-19 Pandemic in the Burden of Hospitalization for Respiratory Syncytial Virus Acute Lower Respiratory Infection in Children under Two Years: A Retrospective Study from Croatia. Viruses. 2022;14(12).

16.	Berikopoulou MM, Dessypris N, Kalogera E, Petridou E, Benetou V, Zahariadou LD, et al. Epidemiology of respiratory syncytial virus in hospitalized children before, during, and after the COVID-19 lockdown restriction measures in Greece. Epidemiol Infect. 2024;152:e94.
